# Nitrogen-Blowing Assisted Strategy for Fabricating Large-Area Organic Solar Modules with an Efficiency of 15.6%

**DOI:** 10.3390/polym16111590

**Published:** 2024-06-04

**Authors:** Yingying Cheng, Yitong Ji, Dongyang Zhang, Xiangda Liu, Zezhou Xia, Xiujun Liu, Xueyuan Yang, Wenchao Huang

**Affiliations:** School of Materials Science and Engineering, Wuhan University of Technology, Wuhan 430070, China

**Keywords:** large-area organic solar modules, power conversion efficiency, morphology control, nitrogen-blowing assisted method

## Abstract

Organic solar cells (OSCs) are one of the most promising photovoltaic technologies due to their affordability and adaptability. However, upscaling is a critical issue that hinders the commercialization of OSCs. A significant challenge is the lack of cost-effective and facile techniques to modulate the morphology of the active layers. The slow solvent evaporation leads to an unfavorable phase separation, thus resulting in a low power conversion efficiency (PCE) of organic solar modules. Here, a nitrogen-blowing assisted method is developed to fabricate a large-area organic solar module (active area = 12 cm^2^) utilizing high-boiling-point solvents, achieving a PCE of 15.6%. The device fabricated with a high-boiling-point solvent produces a more uniform and smoother large-area film, and the assistance of nitrogen-blowing accelerates solvent evaporation, resulting in an optimized morphology with proper phase separation and finer aggregates. Moreover, the device fabricated by the nitrogen-blowing assisted method exhibits improved exciton dissociation, balanced carrier mobility, and reduced charge recombination. This work proposes a universal and cost-effective technique for the fabrication of high-efficiency organic solar modules.

## 1. Introduction

Organic solar cells (OSCs) have garnered substantial attention in industry and academic fields because of their cost-effectiveness, lightweight, facile processing, and flexibility [1,2,3,4,5,6]. High efficiency and upscaling are two key components for the commercialization of this emerging technology [7,8]. Since the star non-fullerene acceptor Y6 has been developed [6], a series of highly efficient derivatives have been designed and synthesized, which has extensively promoted the progress of OSCs [9,10]. With the advancements in these active layer materials and fabrication processes, the power conversion efficiency (PCE) of small-area OSCs is approaching 20% [11,12,13,14]. Nonetheless, the highest efficiency of large-area organic solar modules remains hovering around 16%, far behind their small-area counterparts. This disparity significantly impedes the commercial viability of this emerging technology [15,16].

Numerous advanced OSCs employ a donor–acceptor bulk heterojunction (BHJ) blend as the active layer. The spontaneous phase separation and self-assembly interpenetration of donor and acceptor components provide more expansive interface for exciton dissociation [17]. Therefore, the photovoltaic performance of OSCs is critically dependent upon the microstructural organization of their active layer [18,19]. A multitude of approaches have been proposed to regulate the BHJ morphology across molecular to macroscopic scales [20,21,22,23]. The chemical structure of the donor and acceptor determines the molecular packing, miscibility between donor and acceptor molecules, and solubility in the solvent. These factors directly influence the formation of the blend’s morphology in BHJ structures, which is essential for optimal performance in OSCs [24].

The optimization of film morphology holds significant potential for enhancing the efficiency of organic solar modules. A variety of pre- and post-treatment techniques have been developed, including solvent choice [25], additives [26], thermal annealing [5,27,28], and multicomponent strategy [29,30,31]. For instance, Son et al. demonstrated a 58.5 cm^2^ organic solar module with a PCE of 14.0% by optimizing the morphology of the active layer through adding the third component P(NDI2OD-T2) in the binary blend of PBDB-T-2F:N3 [30]. Chen et al. presented a quaternary strategy with the active layer of PM6:L8-BO:BTP-S10:BTP-eC9 to form the alloy-like phase morphology and enhance the phase homogeneity of donor materials, yielding the PCE of 12.2% with an area of 72.2 cm^2^ [29]. In addition, layer-by-layer deposition was also employed to control the vertical morphology. An organic solar module based on a bilayer of PM6 and Y6 achieves a PCE of 11.86% with an area of 11.52 cm^2^ [16].

However, most high-efficiency organic modules have been fabricated with a low-boiling-point solvent chloroform, which may cause problems of uniformity and reproducibility. Therefore, employing high-boiling-point solvents as an alternative to chloroform is critical for broadening the processing window in the production of organic solar modules. Due to the slow evaporation rate, the resulting films processed with the high-boiling-point solvent usually exhibit unfavorable phase separation, which leads to poor exciton dissociation efficiency [32]. Therefore, it is critical to devise a facile and affordable method to suppress the phase separation and over-size aggregation in the active layer by manipulating the solvent drying rate.

Recently, a novel strategy involving high-temperature solution processing with high-boiling-point solvents has been adopted to manipulate the film-formation kinetics for optimizing the morphology of the active layer [33]. Regulating the flow rate of ambient gases may be another strategy to suppress the phase separation in the active layer. The utilization of air-blowing-assisted techniques has also been developed to facilitate the solvent evaporation rate [34,35]. G. Lidzey et al. utilized an air knife to rapidly remove solvents from the wet films cast by spray coating, thereby enhancing the PCE of PM6:DTY6-based OSCs up to 14.1% [36].

In this study, we develop a nitrogen-blowing assisted approach for developing large-area organic solar modules based on a high-boiling-point solvent chlorobenzene. The nitrogen-blowing assisted method will accelerate the solvent evaporation of active layers, resulting in the fabrication of a uniform and reproducible large-area film with suppressed phase separation. A 5 × 5 cm^2^ PM6:BTP-eC9-based organic solar module is fabricated through the nitrogen-blowing assisted approach. This resulted in a noteworthy PCE of 15.6%, which can be attributed to more efficient exciton dissociation, balanced carrier mobility, and reduced charge recombination. According to our current understanding, this constitutes one of the highest values among all organic solar modules. This work proposes a facile and universal strategy for the morphological manipulation of large-area active layers, promoting the upscaling of organic solar cells.

## 2. Materials and Methods

### 2.1. Materials

PM6, BTP-eC9, PY-IT, PTB7-Th, PC_71_BM and N,N′-Bis{3-[3-(Dimethylamino)propylamino]propyl}perylene-3,4,9,10-tetracarboxylic diimide (PDINN) were sourced from Solarmer Materials Inc., Beijing, China. Poly(3,4-ethylene dioxythiophene):Poly (styrene sulfonate) (PEDOT:PSS) aqueous solutions (Clevios P VP 4083) were purchased from Heraeus, Hanau, Germany. The other solvents were utilized without additional purification after being acquired from Aladdin (Shanghai, China) and Sigma-Aldrich (St. Louis, MI, USA).

### 2.2. Device Fabrication

All OSCs were fabricated in the structure of ITO/PEDOT:PSS/active layer/PDINN/Ag. The pre-patterned indium tin oxide (ITO) glass substrate was cleaned in water, ethanol, and isopropanol for 15 min and dried under N_2_. Next, the ITO substrate was covered with a PEDOT:PSS solution, which had a thickness of approximately 30 ± 10 nm. The substrates were placed in a glovebox after the PEDOT:PSS layer was baked for 15 min in the air at 150 °C. The photoactive layer solutions were prepared by dissolving the PM6:BTP-eC9 (18.7 mg/mL with 0.5 vol.% 1,8-diiodooctane (DIO)), PM6:PY-IT (16.0 mg/mL with 2 vol.% 1-chloronaphthalene (CN)) and PTB7-th:PC_71_BM (20 mg/mL with 2 vol.% DIO), with the weight ratios of 1:1.2, 1:1 and 1:1.2 in chlorobenzene. The active layer solution was spin-coated with a thickness of 100 nm. For the conventional spin-coated samples, the solution was spin-coated at 4000 rpm for 40 s, while for the nitrogen-blown samples, a nitrogen gas stream was introduced at varying pressures (from 0.2 MPa to 0.8 MPa) as a function of delay time (4 s, 6 s, 8 s, 10 s, and 12 s) after the initiation of the spin-coating process. In the experiment, a gas blow gun with a nozzle diameter of 16 mm was positioned above the substrate at a distance of 50 mm. Then, the PDINN was spin-coated at 3000 rpm for 40 s after being dissolved in methanol (0.8 mg/mL). Finally, Ag (110 nm) was thermally evaporated at a vacuum pressure of approximately 1 × 10^−4^ Pa to form electrodes. For large-area organic solar modules, 5 × 5 cm^2^ ITO was pre-patterned into multiple 7 mm wide rectangles. After PEDOT:PSS/active layer/PDINN were deposited, the organic layers were scribed by a 532 nm nanosecond laser beam for subsequent serial interconnection of subcells. Finally, a 100 nm thick Ag electrode was fabricated through vacuum evaporation and shadow masking to finalize the module assembly, thus establishing the interconnection between neighboring cells at an ITO-Ag interface under a pressure of 1 × 10^−4^ Pa.

### 2.3. Characterization

The current–voltage (*J*−*V*) measurements were conducted via the solar simulator (SS-X50, Enlitech, Shanghai, China) under standard AM 1.5G spectral irradiance conditions. The intensity of the solar simulator was calibrated against a certified silicon reference solar cell (SRC2020, Enlitech) to ensure a consistent illumination of 100 mW/cm^2^. The external quantum efficiency (EQE) spectra were measured using a dedicated solar-cell spectral response evaluation system (QE-R, Enlitech). The absorption and transmission spectra were measured with an ultraviolet spectrometer (UV-1900i, Shimadzu, Japan). Film thickness was measured by a Dektak XT probe profiler (produced by Bruker, Billerica, MA, USA). Atomic force microscopy (AFM) images were measured on an atomic force microscope (Cypher ES, Asylum Research, High Wycombe, UK). Transient photocurrent (TPC) and transient photovoltage (TPV) were measured using a transient photocurrent and photovoltage measurement system (LST-TPC, Shanghai Jinzhu Technology Co., Ltd., Shanghai, China). The grazing incident wide-angle X-ray scattering (GIWAXS) measurements were conducted on a Xeuss 3.0 SAXS/WAXS (Xenocs, Grenoble, France) benchtop beamline, utilizing a copper X-ray source operating at an energy of 8.05 keV, coupled with an Eiger 2R 1M detector for data acquisition.

## 3. Results

### 3.1. Device Performance

**Figure 1:**
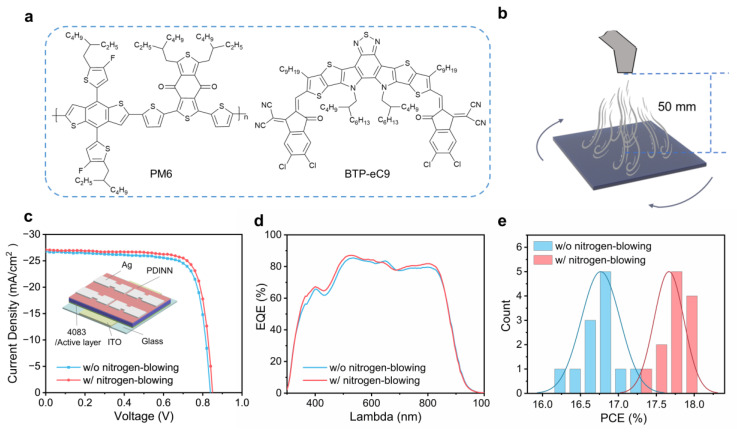
(**a**) Chemical structures of PM6 and BTP-eC9. (**b**) The schematic diagram of the nitrogen-blowing assisted method. (**c**) *J*−*V* curves. (**d**) External quantum efficiency (EQE) spectra. (**e**) Efficiency histograms for PM6:BTP-eC9-based OSCs.

The chemical structure of donor and acceptor materials utilized in this work are shown in Figure 1a. The UV–vis absorption spectra of pure donor and acceptor films are presented in Figure S1. PM6:BTP-eC9 blends display well-matched absorption in the region from visible to near-infrared. The scheme of the nitrogen-blowing assisted method is illustrated in Figure 1b, in which the drying of the active layer is accelerated. Chlorobenzene is selected as the solvent due to its higher boiling point, which can significantly broaden the processing window and improve the uniformity of large-area active layer films [37]. In Figure S2, The UV–vis absorption spectra are exhibited for blend films prepared with and without the nitrogen-blowing assisted method. In the PM6:BTP-eC9 film, the nitrogen-blowing assisted process induced a blue shift in the absorption peak at 700–900 nm compared to untreated films, suggesting the effective suppression of the unfavorable aggregation in the blends processed with high-boiling-point solvents [38,39].

The photovoltaic performance of OSCs produced using the nitrogen-blowing assisted strategy is investigated. The device structure of ITO/PEDOT:PSS/active layer/PDINN/Ag is presented in Figure 1c, associated with the corresponding energy level diagram (Figure S3). The *J*−*V* characteristic of best-performing single-junction OSCs is illustrated in Figure 1c, and their photovoltaic parameters are summarized in Table 1. The control OSCs exhibit a PCE of 17.2% with a *J_SC_* of 26.7 mA/cm^2^, a *V_OC_* of 0.839 V, and an FF of 76.8%, while the OSCs fabricated from the nitrogen-blowing assisted method exhibit the increased *J_SC_* of 27.1 mA/cm^2^, *V_OC_* to 0.848 V and FF to 78.1%, culminating in a boosted PCE of 17.9%. The OSC device performance as a function of the delay time between the initiation of the spin-coating and the implementation of nitrogen-assisted blowing is depicted in Figure S4. In the initial 4 s of the coating process, an excessive amount of residual solvent molecules are still trapped in the film. The introduction of nitrogen-assisted blowing at the early stage leads to increased film thickness and roughness, exerting negative effects on device performance. After 10 s of coating, most of the solvent has already been evaporated, and thus the introduction of nitrogen-blowing has little effect on the evolution of morphology. After the optimization, OSCs processed with a delay time of 6 s exhibit the highest PCE. Moreover, the nitrogen pressure is optimized, and the best pressure is 0.6 MPa (Figure S5).

Figure 1d presents external quantum efficiency (EQE) spectra of OSCs processed with and without the nitrogen-blowing assisted method. The OSCs processed with the nitrogen-blowing assisted method reveal the enhanced EQE across the whole visible region from 400 to 850 nm. The calculated *J_SC_* values integrated from EQE spectra are 26.1 mA/cm^2^ and 25.6 mA/cm^2^ in the device processed with and without the nitrogen-blowing assisted method, respectively. The discrepancies between these *J_SC_* values computed from *J*−*V* characteristics and those derived from the integrated EQE data remain within a 5% margin. In addition, Figure 1e illustrates the statistical analysis of OSC performance under various processing conditions, confirming the superiority of the nitrogen-blowing assisted method.

To assess the universality of this new strategy, the nitrogen-blowing assisted method is also utilized to fabricate fullerene-based and all-polymer solar cells. The utilization of the nitrogen-blowing assisted method shows improved device efficiencies from 14.5% to 15.6% in the PM6:PY-IT device and 8.6% to 9.3% in the PTB7-Th:PC_71_BM device, respectively. (Figures S6 and S7 and Tables S3 and S4).

### 3.2. Device Physics

**Figure 2:**
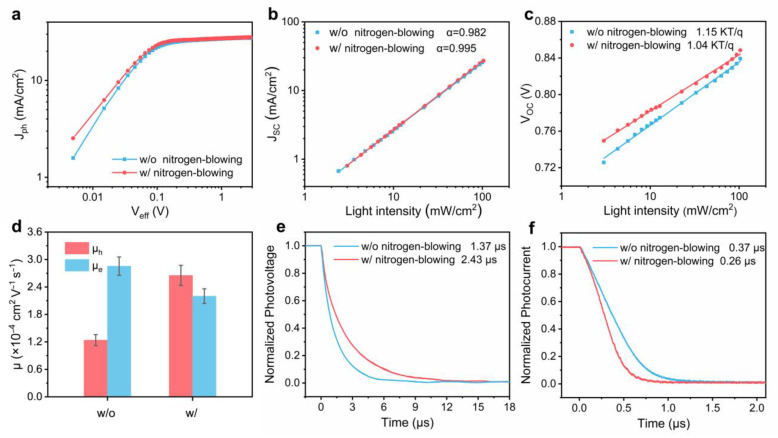
(**a**) Photogenerated current density as a function of effective photovoltage. (**b**) *J_SC_* as a function of light intensity. (**c**) *V_OC_* as a function of light intensity. (**d**) Electron and hole mobilities of OSCs processed with and without the nitrogen-blowing assisted method. (**e**) Normalized transient photovoltage (TPV) decay measurement. (**f**) Normalized transient photocurrent (TPC) decay measurement.

The device physics, such as exciton dissociation and charge collection in OSCs, is studied in Figure 2. The characteristics of exciton dissociation (*η_diss_*) and charge collection (*η_coll_*) are analyzed by plotting the photocurrent density (*J_ph_*) against effective photovoltage (*V_eff_*), plotted in Figure 2a [40]. Here, *J_ph_* is calculated as the difference between *J_L_* and *J_D_*, where *J_L_* represents the illuminated current density, and *J_D_* represents the dark current density. Similarly, *V_eff_* = *V_0_*−*V_appl_*, where *V_0_* is the voltage at *J_ph_* = 0, and *V_appl_* is the applied bias voltage. *J_ph_* observed at elevated *V_eff_* is denoted as the saturated photocurrent (*J_sat_*), premised on the assumption that all light-induced excitons have been completely dissociated into free charge carriers and efficiently collected by the electrodes [41]. The relevant calculations are presented in Table 2. Accordingly, *η_diss_* values are 97.8% and 98.2% for the devices processed without and with the nitrogen-blowing assisted strategy, respectively. The *η_coll_* values of the devices processed without and with treatment are 86.8% and 88.8%, respectively. This indicates that the device processed using the nitrogen-blowing assisted method shows better exciton separation and charge collection.

The charge carrier recombination behavior is assessed by measuring light-intensity-dependent *J_SC_* and *V_OC_*. In Figure 2b, the slope (α) of the curve represents the recombination factor, where a value of 1 signifies no loss of photocurrent in bimolecular recombination [42]. The value of α for OSCs with the nitrogen-blowing assisted method being 0.995 surpasses the value of 0.982 in control devices, suggesting the suppressed bimolecular recombination. Furthermore, the correlation between *V_OC_* and *P_light_* is measured to determine the mode of charge recombination. As shown in Figure 2c, the fitted results for OSCs processed with and without the nitrogen-blowing assisted method are 1.04 KT/q and 1.15 KT/q, respectively. The results demonstrate a decreased trap-assisted recombination in the device processed with the nitrogen-blowing-assisted method.

Hole mobility (*μ_h_*) and electron mobility (*μ_e_*) are determined through the space charge limiting current (SCLC) method, with the results depicted in Figure 2d and detailed in Figure S8. The OSCs processed without the nitrogen-blowing assisted strategy exhibit a considerably elevated electron mobility (*μ_e_* = 2.86 × 10^−4^ cm^2^ V^−1^ s^−1^) in comparison to their hole mobility (*μ_h_* = 1.24 × 10^−4^ cm^2^ V^−1^ s^−1^). The devices exhibit unbalanced carrier mobility, so that charge accumulation at the interface will lead to severe charge recombination. Impressively, more balanced mobility with the hole mobility of 2.65 × 10^−4^ cm^2^ V^−1^ s^−1^ and electron mobility of 2.20 × 10^−4^ cm^2^ V^−1^ s^−1^ is exhibited for OSCs with the nitrogen-blowing assisted strategy.

Carrier lifetime and charge extraction time of OSCs are quantitatively investigated using transient photovoltage (TPV) and transient photocurrent (TPC) decay measurement, as illustrated in Figure 2e,f. The photovoltage decay lifetime of the OSCs processed with the nitrogen-blowing assisted treatment is 2.43 µs, significantly longer than the control device with a decay time of 1.37 µs. The longer decay time suggests the reduced charge recombination. In addition, photocurrent decay times fitted with exponential decay in OSCs processed with and without the nitrogen-blowing assisted method are 0.26 and 0.37 µs, respectively. The faster photocurrent decay time suggests heightened charge extraction efficiency. The synergistic effect of enhanced charge extraction and reduced charge recombination has resulted in a superior FF and *J_SC_* in OSCs prepared with the nitrogen-blowing assisted method.

Band tails of OSCs are assessed using FTPS-EQE, and Urbach energy (*Eu*) is determined by fitting Urbach’s rule to quantify energy disorder, as depicted in Figure S10. The OSCs prepared using the nitrogen-blowing assisted method exhibit a lower energy disorder, with an *Eu* of 21.37 meV, while the control OSCs exhibit an *Eu* of 24.63 meV. The energy disorder is largely affected by the optimized morphology, which will be discussed in the next section [43,44].

### 3.3. Film Morphology

**Figure 3:**
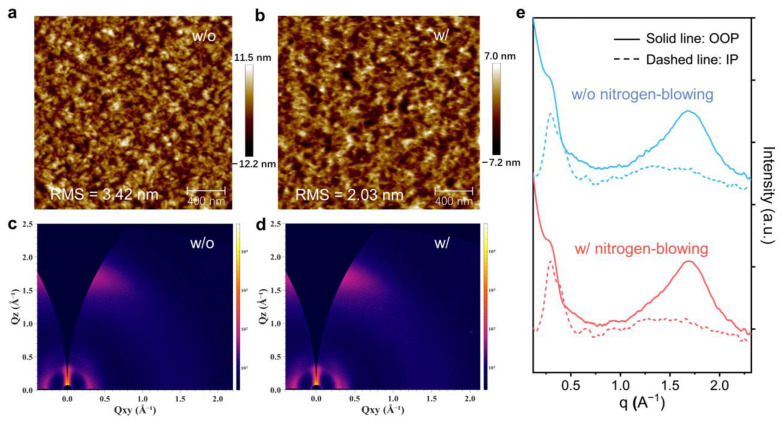
Atomic force microscopy (AFM) height image of PM6:BTP-eC9 films processed (**a**) without and (**b**) with the nitrogen-blowing assisted method. Two-dimensional grazing incidence wide-angle X-ray scattering (GIWAXS) diffraction patterns of PM6:BTP-eC9 films processed (**c**) without and (**d**) with the nitrogen-blowing assisted method. (**e**) Out-of-plane (OOP, solid lines) and in-plane (IP, dash lines) line-cut profiles of the blend films.

To elucidate the improved performance mechanisms, the morphology of active layers with different treatments is compared. Atomic force microscopy (AFM) images demonstrate the surface morphology of the blend films (Figure 3a,b). The blend films exhibit uniform morphology, indicative of excellent compatibility between the donor and acceptor components [45]. However, the fabrication of PM6:BTP-eC9 film layers in the absence of the nitrogen-blowing assistance leads to a heightened root mean square (RMS) surface roughness, quantified at 3.42 nm, as well as the enlarged phase-separated regions with larger aggregate sizes. The unfavorable phase separation exerts a negative effect on exciton dissociation, leading to the lower FF and *J_SC_* of OSCs. In contrast, the films processed with the nitrogen-blowing assisted method exhibit diminished surface roughness (RMS = 2.03 nm), associated with a fine-length scale morphology. Moreover, a uniformly distributed nanoscale phase separation is also obtained, facilitating efficient charge transport and exciton dissociation.

The molecular stacking and crystal orientation in blend films are investigated using grazing-incidence wide-angle X-ray scattering (GIWAXS). The two-dimensional (2D) GIWAXS patterns of active layers are shown in Figure 3c,d, while Figure 3e presents the corresponding 1D profiles along out-of-plane (OOP) and in-plane (IP) directions. All films exhibit a face-on configuration with π–π stacking peak located at the out-of-plane direction. This face-on configuration facilitates charge transport across the active layer [46]. Both films exhibit (100) laminar peaks at 0.30 Å^−1^ (PM6) and 0.40 Å^−1^ (BTP-eC9), suggesting that nitrogen-blowing shows negligible effects on the orientation of the molecular stacking. Regarding the OOP (010) peak corresponding to π–π stacking, the broad peak can be fitted into two distinct contributions: (010) peak of PM6 and (010) peak of BTP-eC9 (Figure S9). The crystal coherence length (CCL) of each peak is calculated using the Scherrer equation (Table S6). The d-spacing of the BTP-eC9 peak diminishes from 3.57 Å to 3.51 Å after the nitrogen-blowing assisted treatment. The shorter distance of the π–π stacking will facilitate the charge transport capabilities. However, the CCL for BTP-eC9 decreases from 36.32 Å to 31.57 Å, indicating that fast solvent evaporation effectively hinders the crystallization of BTP-eC9. The disruption of high crystallization in BTP-eC9 materials can effectively suppress the severe phase separation between donor and acceptor. Interestingly, the crystallization behaviors of donor material PM6 show negligible change after the nitrogen-blowing. Therefore, the application of the nitrogen-blowing assisted method during processing lead to a notable balance in electron and hole mobilities within the active layer, which is also confirmed by SCLC measurement.

### 3.4. Large-Area Organic Solar Modules

**Figure 4:**
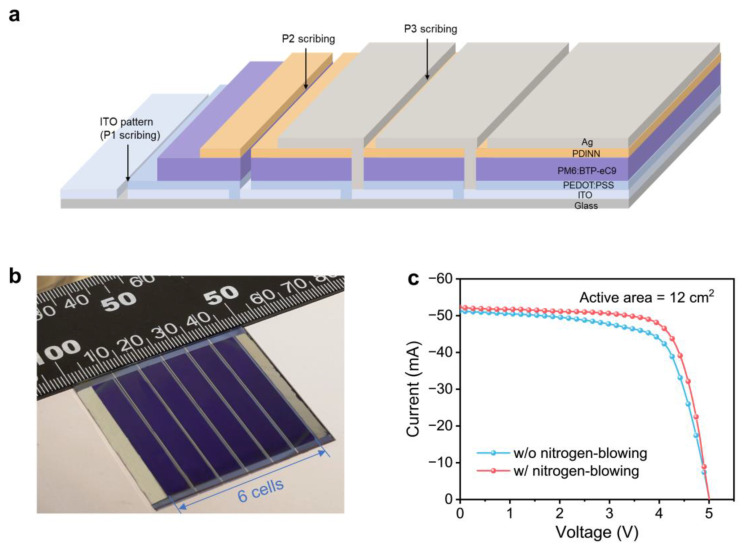
(**a**) Device structure of large-area organic solar modules. (**b**) Photo of the organic solar module containing 6 sub-cells. (**c**) *J*−*V* curves for large-area organic solar modules based on the active layer of PM6:BTP-eC9.

The nitrogen-blowing assisted technique offers great potential as a method for preparing large-area modules. Utilizing the most efficient PM6:BTP-eC9 system as the active layer, a 5 cm × 5 cm organic solar module is designed featuring the following stacked structure: glass/ITO/PEDOT:PSS/PM6:BTP-eC9/PDINN/Ag. The module consists of six sub-cells connected in series, as depicted in Figure 4a,b. In the fabrication of organic solar modules, P1 is patterned by the ITO producer, P2 is scribed by using a nanosecond laser, and P3 is patterned through thermal evaporation by using a shadow mask. The photovoltaic performance of organic solar modules is measured under one sunlight using a mask with an area of 12 cm^2^, and the *J*−*V* curve is shown in Figure 4c. The relevant photovoltaic parameters are summarized in Table 3. The as-cast organic solar module exhibits a PCE of 14.5% with a *V_OC_* of 4.99 V and an FF of 68.2%. The solar module fabricated with the nitrogen-blowing assisted method shows an improved PCE of 15.6% with a *V_OC_* of 5.01 V and an FF of 71.8%. In addition, the modules fabricated using the nitrogen-blowing assisted method also show a higher reproducibility in statistical PCE histograms (Figure S11).

## 4. Conclusions

In summary, a feasible and universal nitrogen-blowing assisted method is developed to control the morphology and uniformity of large-area active layers. By fine-tuning the processing conditions, PM6:BTP-eC9-based OSCs processed by the nitrogen-blowing assisted method reach a PCE of 17.9%, exhibiting superior performance over control devices with a PCE of 17.2%. The improved PCE is attributed to a better phase separation scale and suppressed charge recombination. In addition, this method has used in the fabrication of organic solar modules, with the champion module (active area: 12 cm^2^) delivering a PCE of 15.6%. This research presents a novel method for manufacturing high-performance large-area organic solar modules.

## Figures and Tables

**Table 1:** Photovoltaic parameters of PM6:BTP-eC9-based OSCs prepared with and without the nitrogen-blowing assisted treatment.

Treatment	*V_OC_* (V)	*J_SC_* (mA/cm^2^)	*J_SC_* (EQE) (mA/cm^2^)	FF (%)	PCE (%)
***w/o***	0.839 (0.833 ± 0.006)	26.7 (26.5 ± 0.4)	25.6	76.8 (75.1 ± 2.1)	17.2 (16.7 ± 0.5)
***w/***	0.848 (0.846 ± 0.003)	27.1 (26.9 ± 0.5)	26.1	78.1 (77.2 ± 1.2)	17.9 (17.6 ± 0.3)

**Table 2:** *J_ph_*, *J_sat_*, and *J_ph_/J_sat_* values for PM6:BTP-eC9-based OSCs prepared with and without the nitrogen-blowing assisted treatment.

Treatment	*J_sat_* (mA/cm^2^)	J_ph_* (mA/cm^2^)	*J_ph_^#^* (mA/cm^2^)	*η_diss_* (%)	*η_coll_* (%)
***w/o***	27.31	26.71	23.73	97.8	86.8
***w/***	27.67	27.17	24.57	98.2	88.8

J_ph_*: Photocurrent density under short-circuit condition. *J_ph_^#^*: Photocurrent density at maximal power point condition. *η_diss_*: The characteristics of exciton dissociation (*η_diss_* = J_ph_*/*J_sat_*). *η_coll_*: The characteristics of charge collection (*η_coll_* = *J_ph_^#^*/*J_sat_*).

**Table 3:** Photovoltaic parameters of PM6:BTP-eC9-based organic solar module (12 cm^2^) prepared with and without the nitrogen-blowing assisted treatment.

Treatment	*V_OC_* (V)	*I_SC_* (mA)	FF (%)	PCE (%)	Active Area (cm^2^)
***w/o***	4.99	51.4	68.2	14.5	12
***w/***	5.01	52.2	71.6	15.6	12

## Data Availability

Data are contained within the article and Supplementary Materials.

## Supplementary Materials

The following supporting information can be downloaded at: https://www.mdpi.com/article/10.3390/polym16111590/s1, Figure S1: Normalized UV–vis absorption spectra of donor and acceptor films. Figure S2: Normalized UV–vis absorption spectra of PM6:BTP-eC9, PM6:PY-IT, and PTB7-Th:PC_71_BM films processed with and without the nitrogen-blowing assisted treatment. Figure S3: Energy level diagram. Figure S4: Device performance of PM6:BTP-eC9-based OSCs devices prepared as a function of delay times between coating and nitrogen-assisted blowing. Figure S5: Device performance of PM6:BTP-eC9-based OSCs devices prepared with the nitrogen-blowing assisted strategy as a function of air pressures. Figure S6: (a) Chemical structure of PM6 and PY-IT. (b) *J*−*V* and (c) EQE curves for PM6:PY-IT-based OSCs. Figure S7: (a) Chemical structure of PTB7-Thand PC_71_BM. (b) *J*−*V* and (c) EQE curves for PTB7-Th:PC_71_BM-based OSCs. Figure S8: (a) Electron and (b) hole mobility of PM6:BTP-eC9-based OSCs processed with and without the nitrogen-blowing assisted treatment. Figure S9: Multi-peaks fitting results of in-plane (100) peaks and out-of-plane (010) peaks. Figure S10: FTPS-EQE spectra of PM6:BTP-eC9-based OSCs processed with and without the nitrogen-blowing assisted treatment. Figure S11: Efficiency histograms of large-area modules prepared with and without the nitrogen-blowing assisted treatment. Table S1: Photovoltaic parameters of OSCs prepared as a function of different delay times between coating and nitrogen-assisted blowing. Table S2: Photovoltaic parameters of OSCs prepared with the nitrogen-blowing assisted strategy as a function of air pressures. Table S3: Photovoltaic parameters of PM6:PY-IT-based OSCs prepared with and without the nitrogen-blowing assisted treatment. Table S4: Photovoltaic parameters of PTB7-Th:PC_71_BM-based OSCs prepared with and without the nitrogen-blowing assisted treatment. Table S5: Hole and electron mobility of PM6:BTP-eC9 devices prepared with and without the nitrogen-blowing assisted treatment. Table S6: Information of GIWAXS results for relevant films.
