# Supplementary material for: Nitrogen-Blowing Assisted Strategy for Fabricating Large-Area Organic Solar Modules with an Efficiency of 15.6%
Source: Polymers (Basel). 2024 Jun 4;16(11):1590. doi: 10.3390/polym16111590 (PMC11174350; doi:10.3390/polym16111590)
Supplement: Supplementary file 1 [file polymers-16-01590-s001.zip › polymers-2983463-supplementary.pdf]

Supplementary Materials

Nitrogen-Blowing Assisted Strategy for Fabricating Large-Area Organic Solar Modules with an Efficiency of 15.6%

Yingying Cheng, Yitong Ji, Dongyang Zhang, Xiangda Liu, Zezhou Xia, Xiujun Liu, Xueyuan Yang * and Wenchao Huang *

School of Materials Science and Engineering, Wuhan University of Technology, Wuhan 430070, China

* Correspondence: xueyuan.yang@whut.edu.cn (X.Y.); wenchao.huang@whut.edu.cn (W.H.)

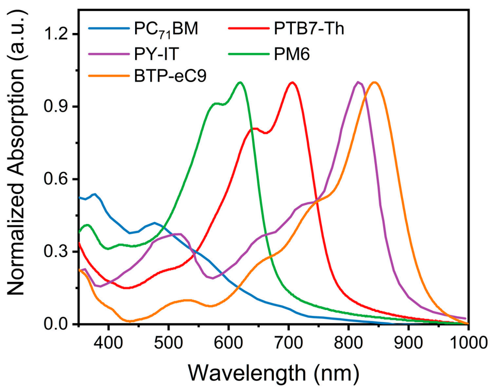

Figure S1. Normalized UV−vis absorption spectra of donor and acceptor films.

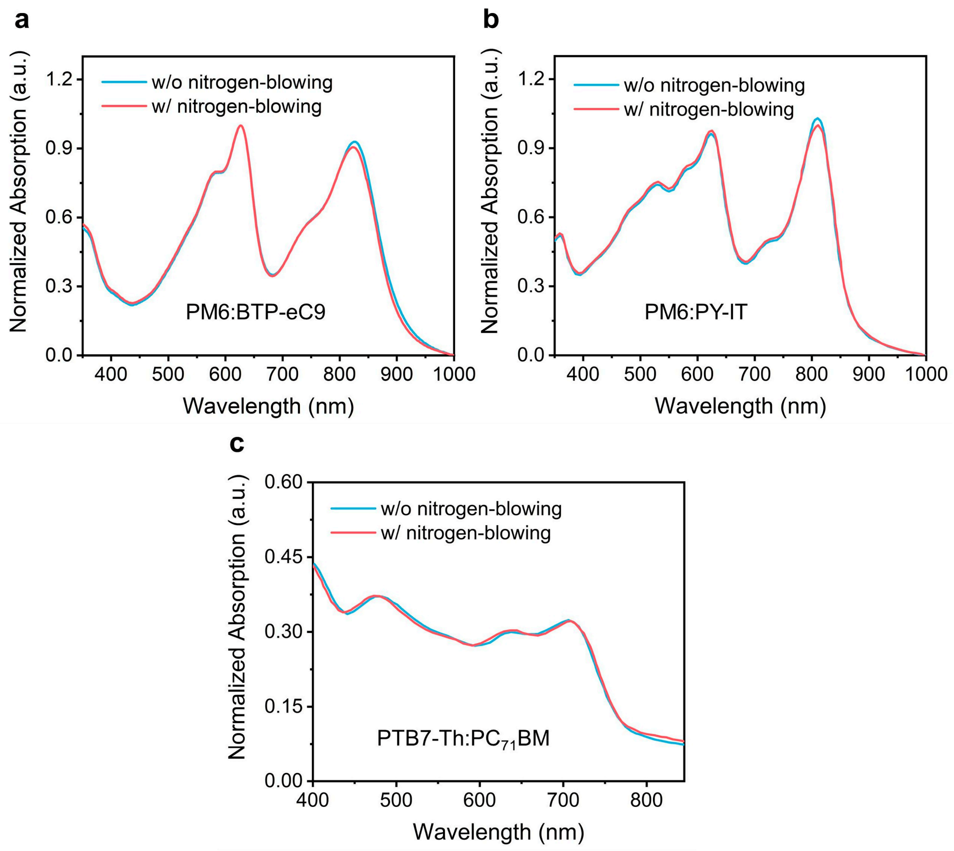

Figure S2. Normalized UV−vis absorption spectra of PM6:BTP-eC9, PM6:PY-IT and PTB7-Th:PC$_{71}$BM films processed with and without the nitrogen-blowing assisted treatment.

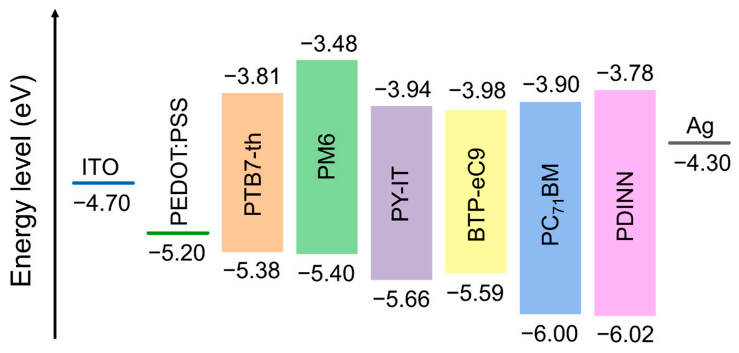

Figure S3. Energy level diagram.

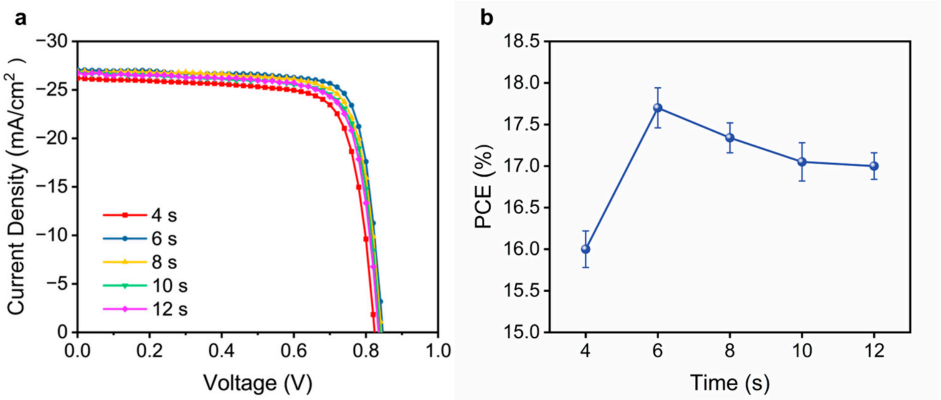

Figure S4. Device performance of PM6:BTP-eC9-based OSCs devices prepared as a function of delay times between coating and nitrogen-assisted blowing.

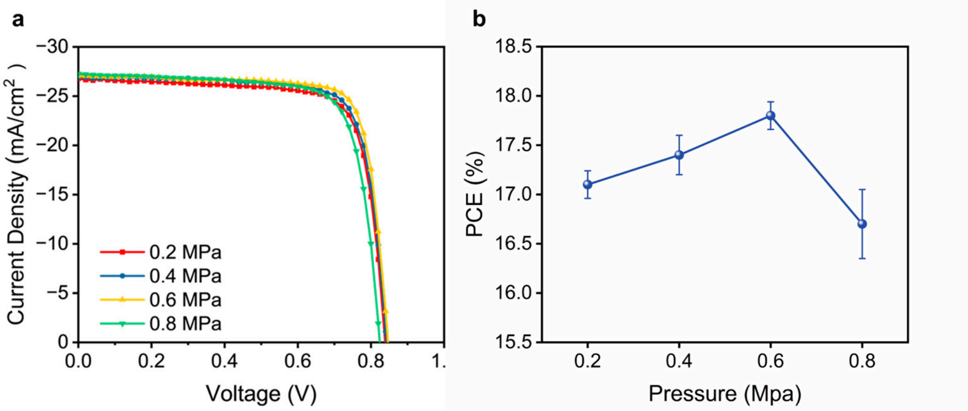

Figure S5. Device performance of PM6:BTP-eC9-based OSCs devices prepared with the nitrogen-blowing assisted strategy as a function of air pressures.

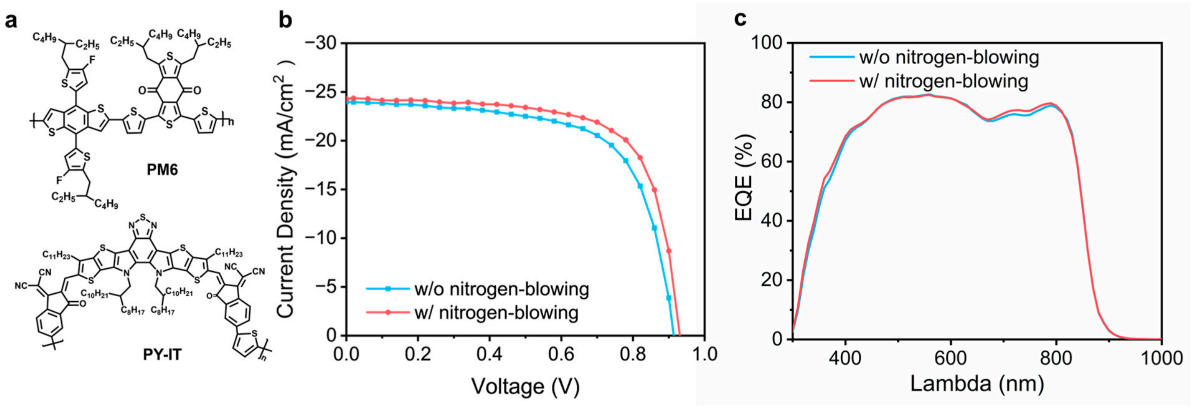

Figure S6. (a) Chemical structure of PM6 and PY-IT. (b) *J*−*V* and (c) EQE curves for PM6:PY-IT-based OSCs.

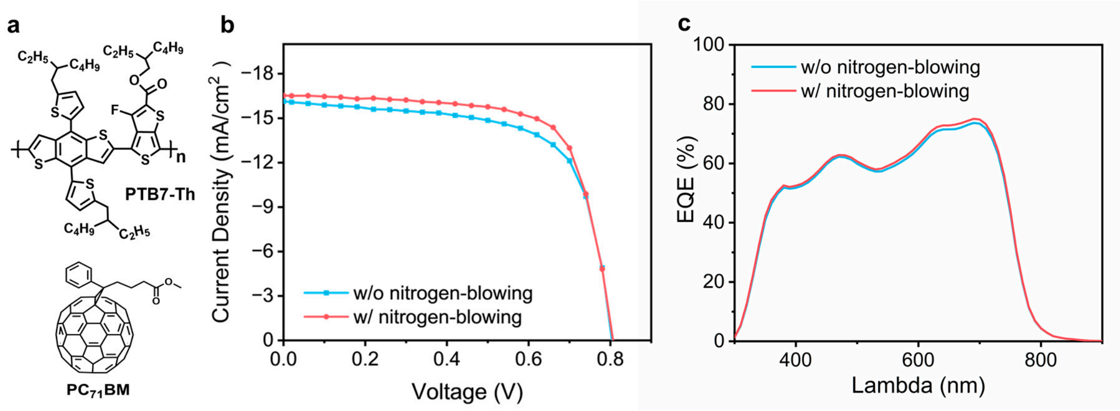

Figure S7. (a) Chemical structure of PTB7-Thand $PC_{71}BM$. (b) *J*−*V* and (c) EQE curves for PTB7-Th:$PC_{71}BM$-based OSCs.

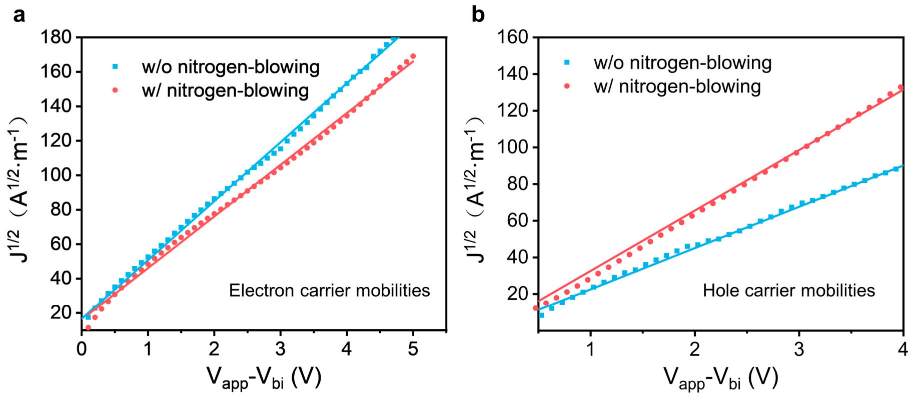

Figure S8. (a) Electron and (b) hole mobility of PM6:BTP-eC9-based OSCs processed with and without the nitrogen-blowing assisted treatment.

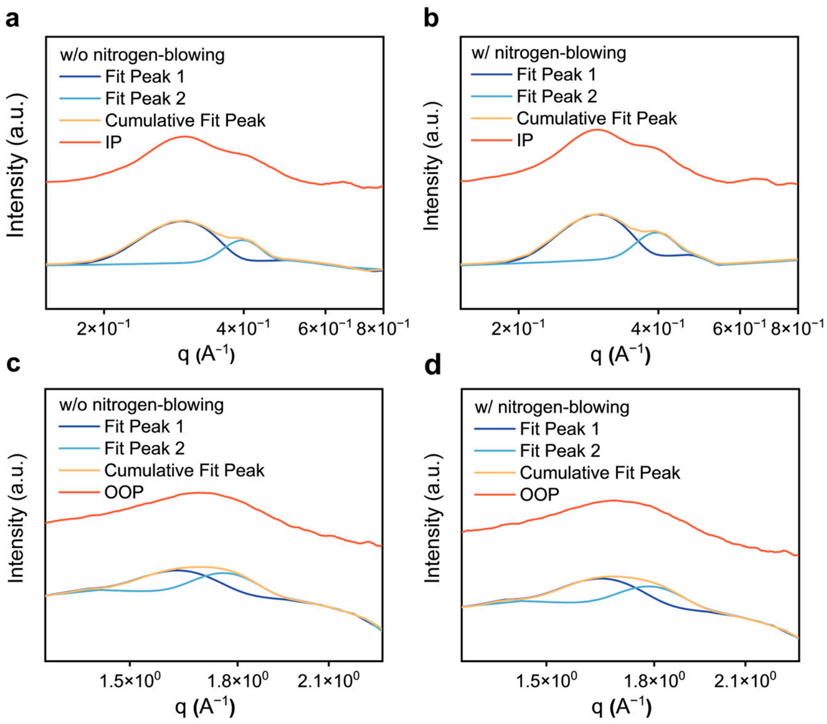

Figure S9. Multi-peaks fitting results of in-plane (100) peaks and out-of-plane (010) peaks.

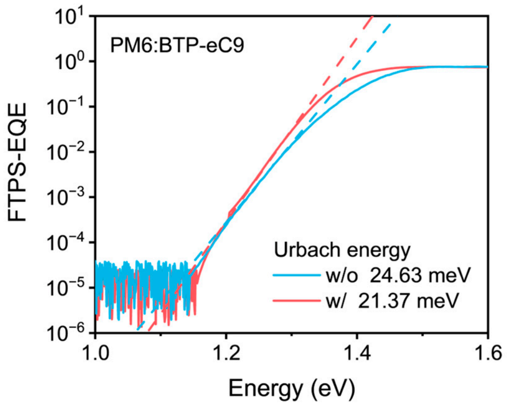

Figure S10. FTPS-EQE spectra of PM6:BTP-eC9-based OSCs processed with and without the nitrogen-blowing assisted treatment.

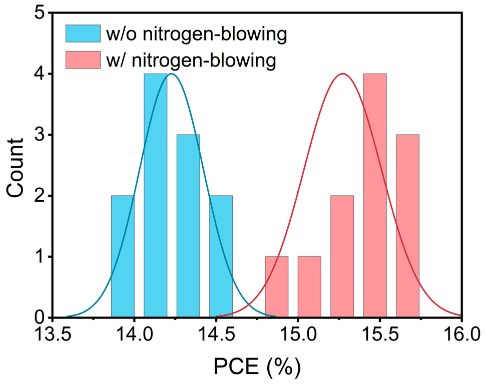

Figure S11. Efficiency histograms of large-area modules prepared with and without the nitrogen-blowing assisted treatment.

Table S1. Photovoltaic parameters of OSCs prepared as a function of different delay times between coating and nitrogen-assisted blowing.

Delay time (s)	V_{OC} (V)	J_{SC} (mA/cm^2)	FF (%)	PCE (%)
4	0.824<br>(0.821±0.004)	26.2<br>(25.9±0.3)	75.0<br>(73.9±1.6)	16.2<br>(16.0±0.2)
6	0.847<br>(0.846±0.003)	27.1<br>(26.9±0.4)	77.9<br>(76.8±1.5)	17.9<br>(17.7±0.2)
8	0.843<br>(0.842±0.003)	26.9<br>(26.7±0.3)	77.1<br>(76.1±2.1)	17.5<br>(17.3±0.2)
10	0.838<br>(0.834±0.004)	26.8<br>(26.4±0.4)	77.0<br>(75.7±1.8)	17.3<br>(17.1±0.2)
12	0.836<br>(0.833±0.003)	26.7<br>(26.3±0.4)	76.9<br>(75.1±2.1)	17.2<br>(17.0±0.2)

Table S2. Photovoltaic parameters of OSCs prepared with the nitrogen-blowing assisted strategy as a function of air pressures.

Pressure (MPa)	V_{OC} (V)	J_{SC} (mA/cm^2)	FF (%)	PCE (%)
0.2	0.838 (0.836±0.003)	26.8 (26.6±0.4)	76.5 (75.9±1.5)	17.2 (17.1±0.1)
0.4	0.841 (0.839±0.004)	26.9 (26.6±0.4)	77.7 (77.0±1.6)	17.6 (17.4±0.2)
0.6	0.846 (0.845±0.003)	27.1 (26.8±0.3)	78.1 (77.1±1.8)	17.9 (17.8±0.1)
0.8	0.823 (0.820±0.004)	27.3 (27.1±0.2)	75.6 (74.3±1.5)	17.0 (16.6±0.4)

Table S3. Photovoltaic parameters of PM6:PY-IT-based OSCs prepared with and without the nitrogen-blowing assisted treatment.

Treatment	V_{OC} (V)	J_{SC} (mA/cm^2)	J_{SC} (EQE) (mA/cm^2)	FF (%)	PCE (%)
w/o	0.928 (0.925±0.004)	23.9 (23.6±0.4)	23.1	65.4 (63.9±1.8)	14.5 (14.2±0.3)
w/	0.939 (0.936±0.005)	24.3 (24.2±0.2)	23.5	68.5 (67.2±1.3)	15.6 (15.4±0.2)

Table S4. Photovoltaic parameters of PTB7-Th:$PC_{71}BM$-based OSCs prepared with and without the nitrogen-blowing assisted treatment.

Treatment	V_{OC} (V)	J_{SC} (mA/cm^2)	J_{SC} (EQE) (mA/cm^2)	FF (%)	PCE (%)
w/o	0.801 (0.799±0.002)	16.1 (15.9±0.3)	15.4	66.8 (64.9±2.0)	8.6 (8.4±0.2)
w/	0.803 (0.800±0.003)	16.5 (16.2±0.3)	15.7	70.2 (69.5±1.8)	9.3 (9.1±0.2)

Table S5. Hole and electron mobility of PM6:BTP-eC9 devices prepared with and without the nitrogen-blowing assisted treatment.

	Hole mobility (μ_h) (cm^{2}V^{-1}s^{-1})	Electron mobility (μ_e) (cm^{2}V^{-1}s^{-1})	μ_h / μ_e
w/o	1.24×10^{-4}	2.86×10^{-4}	0.43
w/	2.65×10^{-4}	2.20×10^{-4}	0.83

Table S6. Information of GIWAXS results for relevant films.

	Samples	q (Å^{-1})	d-spacing (Å)	FWHM (Å^{-1})	CCL (Å)
(IP)	*w/o*	0.30	21.26	0.09	70.67
		0.40	15.77	0.07	89.28
	w/	0.30	21.16	0.08	77.25
		0.40	15.84	0.07	90.35
(OOP)	*w/o*	1.64	3.82	0.21	30.21
		1.76	3.57	0.17	36.32
	w/	1.64	3.81	0.19	32.22
		1.79	3.51	0.20	31.57
